# Myocardial Hypertrophy and Fibrosis Are Associated with Cardiomyocyte Beta-Catenin and TRPC6/Calcineurin/NFAT Signaling in Spontaneously Hypertensive Rats with 5/6 Nephrectomy

**DOI:** 10.3390/ijms22094645

**Published:** 2021-04-28

**Authors:** Evdokia Bogdanova, Olga Beresneva, Olga Galkina, Irina Zubina, Galina Ivanova, Marina Parastaeva, Natalia Semenova, Vladimir Dobronravov

**Affiliations:** 1 Research Institute of Nephrology, Pavlov University, Saint Petersburg 197022, Russia; evdokia.bogdanova@gmail.com (E.B.); beresnevaolga@list.ru (O.B.); ovgalkina@mail.ru (O.G.);zubina@list.ru (I.Z.); biochemlab.pspbgmu@gmail.com (M.P.); 2 Laboratory of Cardiovascular and Lymphatic Systems Physiology, Pavlov Institute of Physiology, Saint Petersburg 199034, Russia; tazhim@list.ru; 3 Research Department of Pathomorphology, Almazov National Medical Research Center, Saint Petersburg 197341, Russia; Natyciel87@gmail.com; 4 Laboratory of Leukemia Research, Russian Research Institute of Hematology and Transfusiology of FMBA of Russia, Saint Petersburg 191024, Russia

**Keywords:** arterial hypertension, cardiac remodeling, chronic kidney disease, calcineurin A, calcineurin B, NFAT, TRPC6, β-catenin, Klotho, fibroblast growth factor 23, parathyroid hormone

## Abstract

Background: Arterial hypertension (AH) is associated with heart and chronic kidney disease (CKD). However, the precise mechanisms of myocardial remodeling (MR) in the settings of CKD remain elusive. We hypothesized that TRPC6, calcineurin/NFAT, and Wnt/β-catenin signaling pathways are involved in the development of MR in the background of CKD and AH. Methods: Early CKD was induced by performing a 5/6 nephrectomy (5/6NE) in spontaneously hypertensive rats (SHR-NE). Sham-operated (SO) SHR (SHR-SO) and Wistar Kyoto (WKY-SO) rats served as controls. Systolic blood pressure (SBP), heart rate, myocardial mass index (MMI), serum creatinine, cardiomyocyte diameter (dCM), myocardial fibrosis (MF), serum and kidney α-Klotho levels, myocardial expression of calcineurin (CaN), TRPC6, and β-catenin were measured two months after 5/6NE or SO. Results: NE-induced kidney dysfunction corresponded to mild-to-moderate human CKD and was associated with an increase in FGF23 and a decrease in renal α-Klotho. The levels of SBP, MMI, dCM, and MF were higher in SHRs compared to WKY-SO as well as in SHR-NE vs. SHR-SO. The MR was associated with increased cardiomyocyte expression of CaN/NFAT and β-catenin along with its intracellular re-distribution. TRPC6 protein levels were substantially elevated in both SHR groups with higher *Trpc6* mRNA expression in SHR-NE. Conclusions: The Wnt/β-catenin and TRPC6/CaN/NFAT hypertrophic signaling pathways seem to be involved in myocardial remodeling in the settings of AH and CKD and might be mediated by FGF23 and α-Klotho axis.

## 1. Introduction

Myocardial remodeling (MR) including cardiomyocyte hypertrophy and interstitial fibrosis is a multifactorial process that develops in response to various pathological stimuli such as pressure overload, neuroendocrine activation, production of paracrine and autocrine factors, as well as impaired energy metabolism [1,2,3].

Ca^2+^ plays a crucial role in regulating MR. In addition to physiological contraction and relaxation of the heart [4], cytoplasmic Ca^2+^ is a second messenger of the intracellular signal transduction pathway responsible for cardiac hypotrophy and fibrosis [5].

An increase in the intracellular concentration of Ca^2+^ is a consequence of its release from intracellular stores and transport through voltage-gated calcium channels of the plasma membrane including the transient receptor potential channels (TRPC) [5]. TRPCs are activated by neuroendocrine factors (catecholamines, angiotensin II) and are involved in the development of arrhythmias, fibrosis and myocardial hypertrophy [5]. In mouse models, overexpression of canonical TRPCs promotes a rapid increase in the concentration of intracellular Ca^2+^ and the activation of one of the main prohypertrophic pathways—the calcium and calmodulin-dependent calcineurin (CaN)/nuclear factor of activated T-cells (NFAT) signaling [6,7,8]. Increased expression of TRPCs is a response to mechanical or oxidative stress [9,10] and NFAT-dependent transcription [11]. It is generally accepted that excessive CaN/NFAT activation and TRPCs overproduction in the heart contribute to the development and progression of pathological MR [1,2,6,7,12,13].

Canonical Wnt/β-catenin signaling pathway (cWnt) is another intracellular signal transduction, which is associated with myocardial hypertrophy [14,15,16,17] and interstitial fibrosis [18,19,20,21] in concert with TGF-β [20,22].

Chronic kidney disease (CKD) is well-known to be an independent cardiovascular risk factor [23]. Arterial hypertension (AH), diastolic dysfunction, and myocardial hypertrophy are highly prevalent among patients with CKD and are associated with increased mortality [23,24,25]. The mechanisms of MR in CKD are poorly understood and may include hemodynamic and non-hemodynamic factors [26,27,28,29,30,31]. The increased production of fibroblast growth factor-23 [32,33], αKlotho [34,35], and calcitriol [36] deficiencies are major candidate non-hemodynamic factors of cardiomyopathy progression in patients with CKD [37,38,39,40].

We hypothesized that CKD has a significant contribution to the development of MR in the background of arterial hypertension, and MR may be mediated by the activation of the above-mentioned intracellular signaling pathways in this case. These results demonstrated that an imbalance in α-Klotho/FGF23, myocardial hypertrophy, and fibrosis are more pronounced and accompanied by the up-regulation of β-catenin and TRPC/CaN/NFAT signaling in the settings of AH and CKD.

## 2. Results

### 2.1. Animal Models of Hypertension and CKD

**Figure 1:**
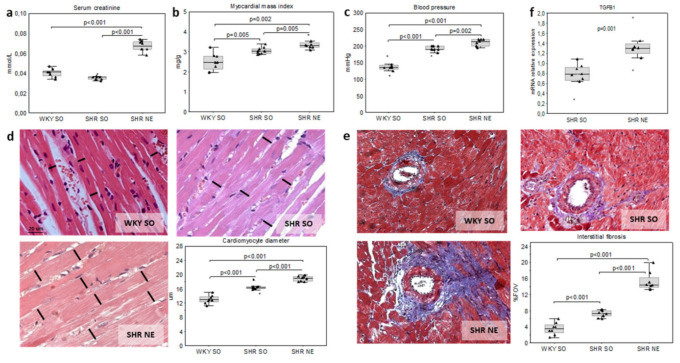
Kidney function and myocardial remodeling in the model of hypertension and chronic renal dysfunction (SHR-NE) and controls (SHR-SO, WKY-SO): (**a**) serum creatinine, (**b**) blood pressure, (**c**) myocardial mass index, (**d**) cardiomyocyte diameter (H&E and quantitative morphometry), (**e**) interstitial fibrosis (Masson’s staining and quantitative morphometry), and (**f**) *Tgfβ1* mRNA relative expression; WKY—Wistar Kyoto rats; SHR—spontaneously hypertensive rats; SO—sham-operated; and NE—nephrectomy.

The systolic blood pressure (SBP) and serum creatinine were expectedly higher in spontaneously hypertensive rats (SHR) with 5/6 nephrectomy (SHR-NE) compared to sham-operated SHR (SHR-SO) and sham-operated Wistar Kyoto rats (WKY-SO) (Figure 1a,c). However, the increase in creatinine in SHR-NE was less than 50% (vs. controls), thus corresponding to mild-to-moderate human CKD [41].

The increase in myocardial mass index (MMI), diameter of cardiomyocytes (dCM), and area of myocardial interstitial fibrosis (IF) were obvious in both SHR groups compared to WKY-SO. The myocardial hypertrophy was more pronounced in SHR-NE versus SHR-SO (Figure 1b,d,e). The greater extent of IF in SHR-NE was associated with the increased *Tgfβ1* gene expression (Figure 1e,f).

The levels of fibroblast growth factor 23 (FGF23) were higher; renal and serum α-Klotho protein were lower in SHRs compared to WKY-SO rats (Table 1). The increase in circulating FGF23 and the decrease in the renal α-Klotho were found in SHR-NE vs. SHR-SO (Table 1). No differences in myocardial expression of Klotho gene and the serum levels of parathyroid hormone (PTH) were found between the groups (Table 1).

### 2.2. Beta-Catenin Myocardial Expression

**Figure 2:**
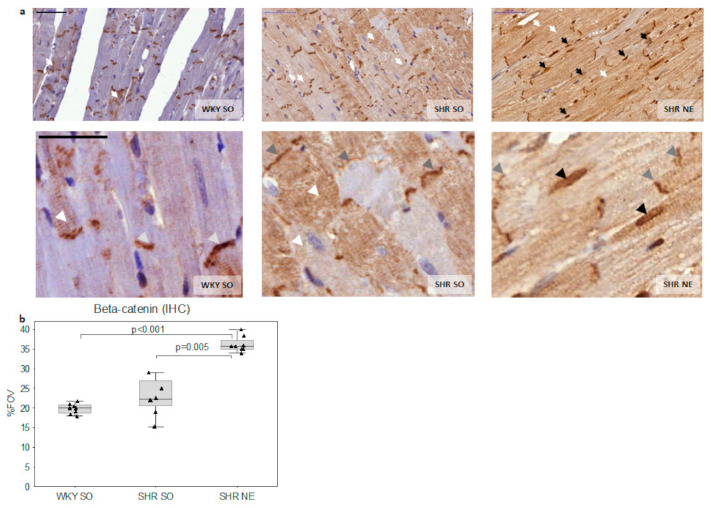
Myocardial expression of β-catenin (IHC): (**a**) representative microphotographs of myocardial β-catenin IHC staining in WKY-SO, SHR-SO, and SHR-NE groups (white arrows—the cytoplasmic expression; grey arrows—intercalated discs; black arrows—the nuclear expression; scale bar, 50 um); (**b**) the total area of myocardial β-catenin expression; WKY—Wistar Kyoto rats; SHR—spontaneously hypertensive rats; SO—sham-operated; NE—nephrectomy; FOV—field of view.

Beta-catenin was localized in intercalated discs, sarcomeres, and cytoplasm of cardiomyocytes in all experimental groups (Figure 2a). The decrease in sarcomere and intercalated discs expression of β-catenin accompanied by the increase in its cytoplasmic expression was obvious in both SHR-SO and SHR-NE groups compared to WKY-SO (Figure 2a). The total area of β-catenin myocardial expression was higher in SHR-NE (Figure 2b) with a high proportion (48.5%) of β-catenin-positive nuclei. Notably, the nuclear expressions of β-catenin were not found in either WKY-SO or SHR-SO groups (Figure 2a). 

### 2.3. CaN/NFAT and TRPC6 Myocardial Expression

**Figure 3:**
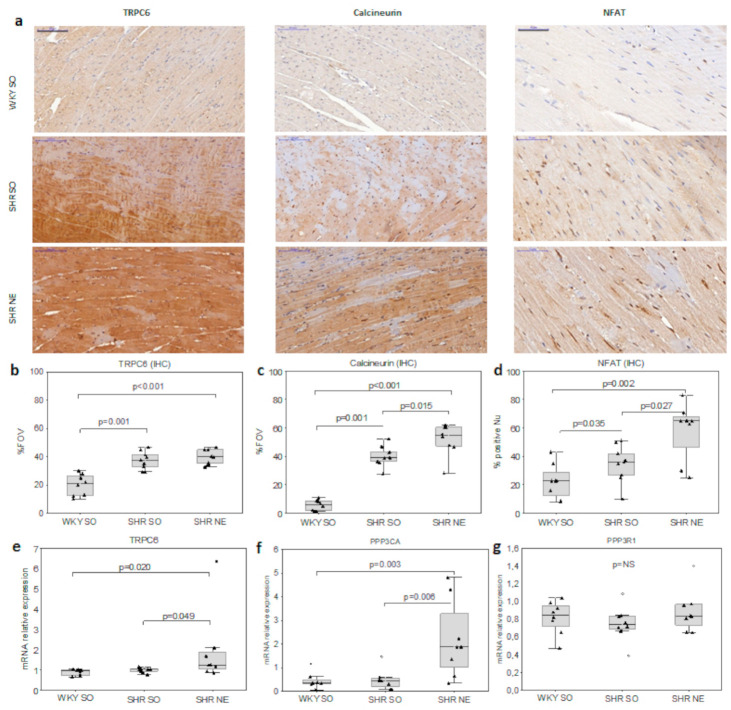
Myocardial expression of calcineurin/NFAT and TRPC6: (**a**) the representative microphotographs of the myocardial expression of TRPC6/CaN/NFAT; the morphometric analysis of myocardial (**b**) TRPC6 (200*×*; scale bar, 100 μm), (**c**) CaN (200*×*), and (**d**) nuclear NFATc3 (400*×*; scale bar, 50 μm) IHC staining; and (**c**) the relative mRNA expression of (**e**) *Trpc6*, (**f**) calcineurin A (*Ppp3ca*) and (**g**) calcineurin B (*Ppp3r1*) in controls, hypertension, and renal dysfunction; FOV—field of view; Nu—nuclei.

Calcineurin and TRPC6 expressions and the number of NFAT-positive nuclei evaluated by IHC were significantly higher in both SHR groups (Figure 3a,b) with the highest CaN/NFAT expression in SHR-NE (Figure 3c,d). The SHR-NE group demonstrated a significant increase in expression of the calcineurin A gene (*Ppp3ca*) and a borderline increase in expression of the *Trpc6*; no increase was seen in the calcineurin B gene (*Ppp3r1*) in myocardium compared to WKY-SO and SHR-SO (Figure 3b).

### 2.4. Interrelations in the Studied Parameters 

In pooled data analysis the myocardial expression of β-catenin positively correlated with dCM (r = 0.74, *p* < 0.05), myocardial fibrosis (r = 0.69, *p* < 0.05), and serum FGF23 (r = 0.57, *p* < 0.05). The inverse association was found between β-catenin and serum renal α-Klotho (r = −0.61, *p* < 0.05). The level of CaN strongly correlated with the number of NFAT-positive nuclei (r = 0.87, *p* = 0.005) as evaluated by IHC. The level of TRPC6 inversely correlated with serum α-Klotho in SHR-NE (r = −0.71, *p* = 0.021). The indices of myocardial hypertrophy and fibrosis were positively associated with CaN/NFAT and TRPC6 IHC expression (Table 2). CaN, NFAT, and TRPC6 inversely correlated with the renal α-Klotho (Table 2) and positively correlated with the serum FGF23 (Table 2).

## 3. Discussion

This proof-of-concept study clearly demonstrated the contribution of CKD to pathological myocardial remodeling in the background of AH. The latter fact concerned the severity of cardiomyocyte hypertrophy and interstitial fibrosis, which was in turn was associated with the up-regulation of the studied signaling pathways: β-catenin, TRPC/CaN/NFAT, and the imbalance in α-Klotho/FGF23 axis.

Previous studies performed in models with intact kidneys showed that cWnt activation in the myocardium occurs during the development of cardiac hypertrophy [15,16] and fibrosis [18,19,20,21]; the inhibition of this signaling pathway has the opposite effect [14,17].

MR processes in experimental CKD are characterized by alterations of cardiomyocyte β-catenin expression. We confirmed earlier observations of a decrease in β-catenin in intercellular contacts and its simultaneous increase in cytoplasm in the model of cardiorenal syndrome in rats with aortic ligation [42]. In addition, the intracellular redistribution of β-catenin was revealed for the first time to be accompanied by its nuclear translocation in the in vivo model of CKD and AH.

Previous studies have shown that the intercalated discs of cardiomyocytes are an important organizing center for various surface proteins including those involved in intercellular interactions. Beta-catenin is a structural protein of the adherens junction of cardiomyocytes and its reduction in the intercalated discs is associated with progressive cardiac hypertrophy and the development of cardiomegaly and heart failure [43]. Changes in the intracellular expression of β-catenin may reflect the pathological rearrangement of the cytoskeleton in response to pressure overload in hypertension [15].

In contrast, an increase in cytoplasmic β-catenin may reflect the activation of intracellular cWnt signaling. The nuclear localization of β-catenin is associated with cWnt up-regulation in various cell types [44,45]. Previous studies have shown the nuclear accumulation of β-catenin in primary culture of cardiomyocytes exposed to angiotensin II [46] and in vivo in rats with spontaneously hypertensive heart failure [15]. Nuclear staining of β-catenin is not observed in hypertension without chronic renal dysfunction and is likely a specific feature of the CKD-related myocardial alterations and an evidence of cWnt activation in cardiomyocytes in vivo. Collectively, these results suggest the involvement of β-catenin in CKD- and AH-induced MR including both cytoskeleton alterations and activation of cWnt signaling.

β-catenin is up-regulated in cardiac fibroblasts and is accompanied by pro-fibrotic gene expression [20,22]. In this context, the increase in *Tgfb1* expression in myocardium may be the consequence of β-catenin-dependent activation of fibrogenesis in a model of combined CKD and AH exposure. 

This study showed an increase in the expression of protein TRPC6 in AH regardless of CKD confirming data about the role of these stress-induced ion channels in the development of MR [8,9,10,11]. TRPCs mediate a fast increase in the cytoplasmic Ca^2+^, which activates calmodulin and CaN/NFAT in cardiomyocytes [8,9,10,13]. The myocardial hypertrophy is associated with activation of the TRPC/CaN/NFATc3 axis in AH models with intact kidneys [7,11,47,48,49], which is confirmed in this study. The increase in CaN expression and the number of NFAT-positive nuclei indicate the activation of CaN/NFAT signaling at the MR induced by the experimental CKD in this study; this further confirms the clinical observations [33].

Canonical TRPC channels are the main link between mechanical or oxidative stress and Ca^2+^ influx into cardiomyocytes that regulate the CaN/NFAT axis [9,10] to explain the co-directional changes in TRPC6, CaN, and NFATc3 as demonstrated in our study. NFAT regulates the transcription of many genes associated with cell differentiation and growth [12]. The *Trpc6* promoter contains NFAT-responsive elements and the borderline increase in *Trpc6* expression as observed in SHR-NE versus SHR-SO and WKY-SO; this is likely mediated by the activation of NFAT [11]. 

The development and progression of CKD is accompanied by changes in circulating hormone levels that may be related to MR. Some of these have been investigated. An increase in FGF23 is typical in CKD in response to the renal phosphate retention [37,38,39,40]. FGF23 was significantly higher in SHR-SO and SHR-NE groups compared to WKY-SO. FGF23 also correlated with MR indices confirming numerous clinical observations of the association of FGF23 and left ventricular hypertrophy [33,50,51,52]. 

The kidneys and parathyroid glands are the classical target organs of the endocrine effects of FGF23 where FGF23, together with the co-receptor α-Klotho, implements its main physiological functions [53]. Circulating FGF23 as well as FGF23 secreted by cardiomyocytes interact with a specific receptor FGFR4 to induce myocardial hypertrophy and fibrosis [32,33,54]. FGF23 is a factor mediating the activation of intracellular Ca^2+^-dependent proteins (PLCγ and CaN/NFAT) [50,55] as well as β-catenin and TGF-β [56]. On the contrary, α-Klotho downregulates the pro-hypertrophic signals of FGF23 [54], growth factors [8,57,58], and Wnt [59]. MR was not associated with myocardial α-Klotho mRNA expression. A decrease in renal and circulating α-Klotho is typical for hypertension and CKD [34,38,39,40], including the models used here. Thus, a decline in α-Klotho kidney production and circulating α-Klotho may be an additional factor in the progression of MR.

The study does have some limitations. First, the applied models correspond to the relatively early stages of hypertension and CKD, and the results cannot be translated to more pronounced stages of these pathological processes. Second, the factors related to kidney dysfunction were limited to renal α-Klotho and circulating α-Klotho, FGF23, and PTH. The other factors do not include the renin–angiotensin–aldosterone system and myocardial expression of FGF23/FGFR, which can be involved in MR, cWnt, and CaN/NFAT alterations in CKD and AH; this requires further study. Third, polyclonal antibodies (Ab) to the N-terminus region of beta-catenin may recognize both the unphosphorylated (active) and the phosphorylated (inactive) isoform of the molecule. Nevertheless, it was expected that most of b-catenin detected by this Ab was likely unphosphorylated by GSK3 [60]. Finally, it was clearly shown that alterations of the signaling pathways were associated with the development of MR in the background of CKD and AH. However, this study was not designed to validate the proposed mechanisms and more future studies are warranted in these areas.

## 4. Conclusions

In conclusion, kidney dysfunction in the background of arterial hypertension and alterations of FGF23/α-Klotho axis has a substantial impact on cardiomyocyte hypertrophy and interstitial fibrosis. Myocardial remodeling in the experimental CKD is associated with activation of β-catenin and TRPC6/CaN/NFAT cardiomyocyte signaling.

The results of the present study may have implications in designing future experimental and clinical research for better understanding the impact of CKD on myocardial remodeling and developing novel therapeutic options in patients with cardiorenal syndromes.

## 5. Materials and Methods

### 5.1. Animals 

Adult male SHR as well as WKY rats weighing 190–230 g were housed using a 12-h/12-h light/dark cycle at room temperature (20–22 °C) and given ad libitum access to water and standard rat chow containing 0.6% phosphate. Early CKD was induced by 5/6 nephrectomy in SHR. Controls were sham-operated WKY and SHR (Table 1). The observation period was two months. SBP was measured before surgery and the day before the experiment via a tail-cuff method using an electrometer (ELEMA, Sweden). In each rat, five measurements of SBP were performed, and the mean value of the last three measurements was calculated. Heart rate was determined by the recorder H-338-2P (Russia) at a paper speed 10 mm/s. Two months after surgery, animals were euthanized by decapitation, and blood and tissue samples were collected. The MMI was calculated as a ratio of the myocardium mass (mg) to the mass of the rat (g).

### 5.2. Laboratory Measurements

The blood samples were centrifuged at 3000 rpm for 10 min, aliquoted, and stored at −80 °C with temperature control for future analysis for less than six months. The stored samples underwent a single thaw followed by assays. Serum creatinine was measured with an enzymatic method using reagent kits on SYNCHRON CX DELTA (Beckman Coulter, USA). The levels of intact FGF23 were measured using a FGF23 ELISA Kit (Kainos Laboratories, Inc., Tokyo, Japan), PTH—using a Rat Intact PTH ELISA Kit (Immutopics, Inc., San Clemente, 92673, USA), and serum α-Klotho ELISA Kit for Rat (Cloud-Clone Corp., Wuhan, China).

### 5.3. Histology and IHC

The heart and kidney were removed immediately after sacrifice and prepared for histological analyses. Next, 2-mm myocardial and renal cortex cross-sections were fixed in 5% neutral buffered formalin for 16 h at room temperature, embedded in paraffin, and cut at one- or two-micron sections. Deparaffinized and rehydrated sections were stained with hematoxylin and eosin (H&E), Masson’s trichrome, and IHC reagents.

*IHC.* After antigen retrieval sections were incubated with rabbit polyclonal antibodies against calcineurin (1:100 dilution, PA529939, Thermo Fisher Scientific, Waltham, MA 02451, USA), TRPC6 (1:100 dilution, PA520256, Thermo Fisher Scientific, Waltham, MA 02451, USA), NFATc3 (1:100 dilution, PA536101, Thermo Fisher Scientific, Waltham, MA 02451, USA), N-term β-catenin (1:200 dilution, GTX101435, GeneTex, Irvine, CA 92606, USA) or α-Klotho (1:200, ab69208, Abcam, Cambridge, UK) for one hour at room temperature followed by Histofine^®®^ Simple StainTM MAX PO I detection system for 30 min at room temperature (Nichirei Biosciences Inc., Tokyo, Japan). A 3,3-diaminobenzidine Histofine^®®^ DAB-3S kit (Nichirei Biosciences Inc., Tokyo, Japan) was used as the chromogen. Finally, the slides were counterstained with hematoxylin and mounted after dehydration.

*Morphometric analyses.* Each parameter was scored quantitatively by a blinded observer who examined 10 fields of view (200× *g* magnification) in one section for each animal using VideoTesT-Master (Morphology) software (VideoTesT, Saint Petersburg, Russia). Twenty measurements of cardiomyocyte diameter were done in each of 10 separate fields of view for one section. The areas of myocardial fibrosis were calculated in 10 fields of view for one section. The area of IHC staining for calcineurin, TRPC6, β-catenin, and α-Klotho (% of the FOV) as well as the number of NFAT-positive nucleus (the ratio of positive to all nuclei in %) were scored in 10 fields of view for one section. The mean values were obtained for each animal, which then were used for statistical analysis (n = 8, for each group).

### 5.4. Real-Time PCR

The myocardium walls one-two mm^3^ samples were incubated in RNA later IntactRNA overnight at 4 °C (Evrogen JSC, Russia) and stored at −80 °C for future analysis. Total RNA was extracted using extraction kits RIBO-zol-C and RIBO-sorb (AmpliSens, Moscow, Russia) following the manufacturer’s instructions and then digested with DNAse I (Thermo Scientific, Waltham, MA 02451, USA). For each sample, 1 μg of total RNA was reverse transcribed using MMLV RT kit (Evrogen JSC, Moscow, Russia). Primers and qPCRmix-HS SYBR (Evrogen JSC, Moscow, Russia) were added to 100 ng of cDNA. The mRNA expression was evaluated by semi-quantitative RT-PCR analysis using the primers for four target and two reference genes (Table 3). The PCR efficiency was assessed for each pair of primers. The amplification program consisted of initial denaturation (95 °C, 3 min) and 40 cycles including denaturation (94 °C, 20 s), primer annealing, and elongation (60 °C, 40 s). The expression of the target gene was calculated by the ΔΔCt method to adjust the PCR efficiency.

### 5.5. Statistical Analysis

Values were expressed as median (interquartile range (IQR)). The groups were compared using a two-sided Mann–Whitney U-test. The association between variables was evaluated by Spearman’s coefficient. Analyses were performed using SAS version 9.4 (SAS Institute Inc., Cary, NC, USA). *p*-values < 0.05 were considered statistically significant.

## Figures and Tables

**Table 1:** Animal models and the investigated parameters.

Parameters	Group 1 WKY-SO	Group 2 SHR-SO	Group 3 SHR-NE
Strain	Wistar Kyoto rats	Spontaneously hypertensive rats	
Model	control	hypertension	hypertension and CKD
Surgery	sham-operated	sham-operated	5/6 nephrectomy
Observation period, mo	2	2	2
Rats number, n	8	8	8
HR, bpm	401 (393–417)	400 (374–416)	412 (397–436)
PTH, ng/mL	96.9 (53.3–137.3)	37.2 (21.5–105.3)	95.3 (80.3–123.0)
FGF23, ng/mL	293.4 (243.0–361.2)	627.6 (383.7–688.9) ^a^	767.5 (701.1–889.9) ^a b^
rKlotho, % FOV	34.2 (29.5–36.0)	25.3 (16.2–28.0) ^a^	13.1 (12.5–14.8) ^c^
sKlotho, ng/mL	2698.3 (2413.3–2830.9)	800.2 (606.6–890.4) ^a^	1310.4 (1115.7–1541.9) ^d^
m*Kl*/*Gapdh, Hprt1*	0.39 (0.31–0.41)	0.35 (0.27–0.40)	0.36 (0.25–0.43)

HR—heart rate; PTH—parathyroid hormone; FGF23—fibroblast growth factor 23; rKlotho—renal α-Klotho protein, determined by IHC; sKlotho—serum α-Klotho protein; m*Kl/Gapdh, Hprt1*—relative expression of α-Klotho mRNA in myocardium; ^a^—*p* ≤ 0.005 compared to WKY-SO, ^b^—*p* ≤ 0.007 compared to WKY-SO and SHR-SO, ^c^—*p* < 0.001 compared to WKY-SO and SHR-SO, and ^d^—*p* ≤ 0.003 compared to WKY-SO and SHR-SO.

**Table 2:** Pooled correlation analyses.

Parameters	TRPC6, %FOV	CaN, %FOV	NFAT, %FOV	b-catenin, %FOV
sKlotho, pg/mL	−0.47 *	−0.37	−0.21	−0.28
rKlotho, %FOV	−0.69 *	−0.82 *	−0.67 *	−0.61 *
FGF23, ng/mL	0.58 *	0.68 *	0.46 *	0.57 *
PTH, ng/mL	−0.27	−0.14	−0.05	0.19
sCr, mmol/L	0.11	0.29	0.22	0.48
MMI, mg/g	0.61 *	0.75 *	0.68 *	0.45
dCM, um	0.74 *	0.84 *	0.74 *	0.74 *
IF, %FOV	0.71 *	0.88 *	0.80 *	0.69 *

sKlotho—serum α-Klotho protein; rKlotho—renal α-Klotho protein; FGF23—fibroblast growth factor 23; PTH—parathyroid hormone; sCr—serum creatinine; MMI—myocardial mass index; dCM—diameter of cardiomyocytes; IF—interstitial fibrosis; TRPC6—transient receptor potential cation channel subfamily C member 6; CaN—calcineurin; NFAT—nuclear factor of activated T-cells; FOV—field of view, * *p* < 0.05.

**Table 3:** Primer sequence for qRT-PCR.

Primer Name	Sequence (from 5′ to 3′)	Accession Number	Gene Product
*Ppp3ca*		NM_017041.2	Protein phosphatase 3 catalytic subunit alpha
Forward	CAGTAACTTTCGAGCCAGCC		
Reverse	GACTTGGCGGAAATGGAACG		
*Ppp3r1*		NM_017309.2	Protein phosphatase 3 regulatory subunit B, alpha
Forward	AGCTTGACTTGGACAACTCT		
Reverse	ATATCTAGGCCACCTACAAC		
*Trpc6*		NM_053559.1	Transient receptor potential cation channel, subfamily C, member 6
Forward	GTGAACGAAGGGGAGCTGAA		
Reverse	GCGGCTTTCCTCTTGTTTCG		
*Tgfb1*		NM_021578.2	Transforming growth factor, beta 1
Forward	TGGCGTTACCTTGGTAACC		
Reverse	GGTGTTGAGCCCTTTCCAG		
*Kl*		NM_031336.2	Klotho (Kl)
Forward	ACTTCGGTGGTCAGGTCAAG		
Reverse	CTCTTTGGGTAGTCGCCATC		
*Gapdh*		NM_017008.4	Glyceraldehyde-3-phosphate dehydrogenase
Forward	AGATGGTGAAGGTCGGTGTG		
Reverse	GATCTCGCTCCTGGAAGATG		
*Hprt1*		NM_012583.2	Hypoxanthine phosphoribosyltransferase 1
Forward	GTTGGATACAGGCCAGACTT		
Reverse	GCCACATCAACAGGACTCTT		

## Data Availability

All data presented in this study are available from the corresponding author on reasonable request.
